# Spin-density studies of the multiferroic metal-organic compound [NH_2_(CH_3_)_2_][Fe^III^Fe^II^(HCOO)_6_]

**DOI:** 10.1107/S205225252000737X

**Published:** 2020-07-17

**Authors:** Laura Cañadillas-Delgado, Oscar Fabelo, J. Alberto Rodríguez-Velamazán, Anne Stunault, Jiong-Peng Zhao, Xian-He Bu, Juan Rodríguez-Carvajal

**Affiliations:** a Institut Laue Langevin, 71 avenue des Martyrs, CS 20156, Grenoble, Cedex 9 38042, France; b Department of Chemistry, and TKL of Metal and Molecule-Based Material Chemistry, Nankai University, Tianjin 300071, People’s Republic of China

**Keywords:** spin density, multiferroics, polarized neutron diffraction, magneto-electric behaviour

## Abstract

Polarized neutron diffraction is used to study in depth the magnetic properties of the heterometallic compound [NH_2_(CH_3_)_2_][Fe^III^Fe^II^(HCOO)_6_] and give insight into its magnetic behaviour, addressing open questions that will contribute to a better understanding of this attention-grabbing material and other related ones. Previous results revealed that upon cooling, the magnetic moments of the Fe^II^ and Fe^III^ sites do not order simultaneously: the magnetization of the Fe^II^ site increases faster than that of the Fe^III^ sites. Unpolarized neutron diffraction measurements at 2 K with no external field revealed some discrepancies in the saturation value of the magnetic signal on the Fe^III^ sites and in the ferromagnetic moment along the *c* axis. These discrepancies could be related to the actual distribution of magnetic moment, since unpolarized neutron diffraction gives information on the magnetic moment localized only on the magnetic ions. Polarized neutron diffraction allows an analysis of the magnitude of the spin density over magnetic and non-magnetic ions (the organic ligand and the counterion), which can give a clue to explain the low saturation on the Fe^III^ sites and the correlation with the physical measurements. The present study also contributes to the understanding of the magneto-electric behaviour of this compound, giving insight into the role of metal disorder in the origin of the structural phase transition, which is responsible for its antiferrolelectric order, and into the influence of spin-density delocalization on its magneto-electric properties, allowing a discussion of the alternative explanations given so far for its electric properties at low temperature.

## Abstract 2

Polarized neutron diffraction on the flipping-ratio technique has been used to determine the spin-density map for the multiferroic compound [NH_2_(CH_3_)_2_][Fe^III^Fe^II^(HCOO)_6_]. The proposed models give an alternative explanation for the origin of the multiferroic behaviour of this compound.

## 1. Introduction   

Metal-organic frameworks combining two or more properties have been the focus of interest of several research groups in the last few decades [see, for example, Thomas *et al.* (1996 ▸), Ruiz *et al.* (1997 ▸), Wang *et al.* (2004 ▸), Zhou *et al.* (2012 ▸), Berg *et al.* (2012 ▸), Cucinotta *et al.* (2012 ▸), Adhikary *et al.* (2014 ▸)]. A good approach to achieve this combination has been the design of perovskite-like metal-organic frameworks where a three-dimensional network made of metallic centres linked through organic ligands, which presents magnetic order, also accommodates counterions responsible for electric order [see, for example, Chitnis *et al.* (2018 ▸), Mączka *et al.* (2017 ▸), Malik *et al.* (2018 ▸), Hughey *et al.* (2018 ▸), Mazzuca *et al.* (2018 ▸), Stroppa *et al.* (2013 ▸), Fu *et al.* (2011 ▸), Jain *et al.* (2009 ▸)]. One of these examples is the mixed-valence iron(II)–iron(III) formate compound [NH_2_(CH_3_)_2_][Fe^III^Fe^II^(HCOO)_6_], (**1**).

**Figure 1:**
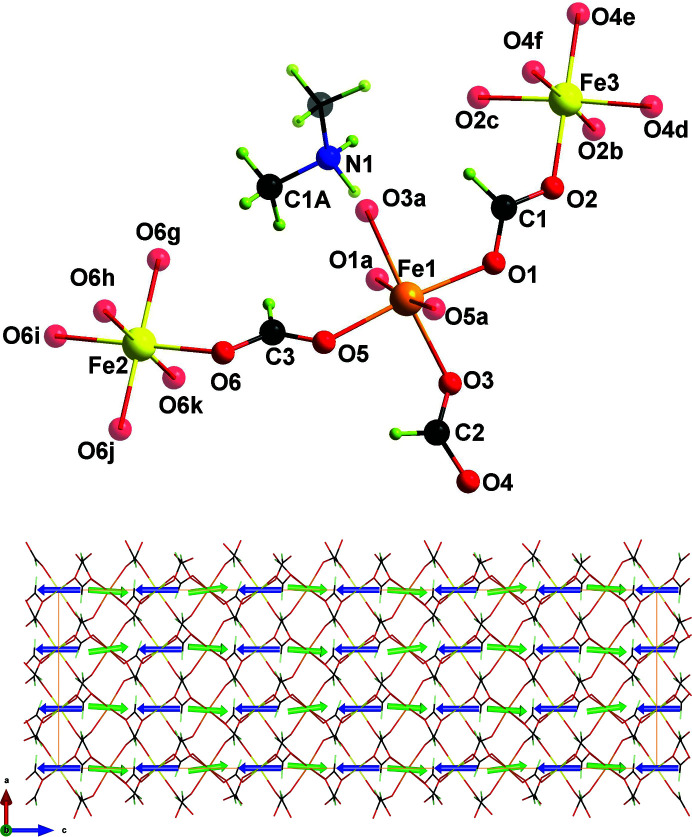
(Top) A view of a fragment of compound (**1**). Solid coloured atoms represent the asymmetric unit. The Fe^III^ atoms (Fe2 and Fe3) are shown in yellow and the Fe^II^ atom (Fe1) is orange. Oxygen, carbon, nitro­gen and hydrogen atoms are displayed in red, black, blue and lime, respectively. (Bottom) A view along the *b* axis of the unit cell of compound (**1**), together with the magnetic moments of the Fe^II^ and Fe^III^ sites (green and blue arrows, respectively) determined at 2 K from neutron diffraction (Cañadillas-Delgado *et al.*, 2012 ▸). [Symmetry codes: (*a*) 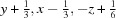; (*b*) 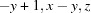; (*c*) 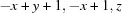; (*d*) 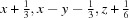; (*e*) 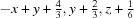; (*f*) 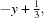 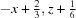; (*g*) 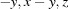; (*h*) 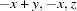; (*i*) 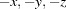; (*j*) 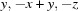; (*k*) 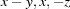.]

The crystal structure and electric and magnetic behaviours of compound (**1**) have been analysed in depth in previous reports (Hagen *et al.*, 2009 ▸; Zhao *et al.*, 2010 ▸; Cañadillas-Delgado *et al.*, 2012 ▸; Ciupa *et al.*, 2015*b* ▸; Guo *et al.*, 2017 ▸; Sieradzki *et al.*, 2016 ▸). It presents a structural phase transition at *ca* 155 K, which involves a change from space group 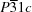 [*a* = *b* = 8.2550 (12) Å and *c* = 13.891 (3) Å] at room temperature to 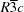 [*a* = *b* = 14.2600 (17) Å and *c* = 41.443 (8) Å] at low temperature. The crystal structure at room temperature consists of a three-dimensional network made of Fe^II^ ions connected to six Fe^III^ atoms by means of six formate ligands in an *anti*–*anti* coordination mode (see Fig. 1 ▸, top), constructing an alternating mixed-valence network. This structural network topology corresponds to a niccolite bimodal network (4^12^ 6^3^) (4^9^ 6^6^), where the Fe^III^ ion lies on the (4^12^ 6^3^) node and the Fe^II^ site is placed on the (4^9^ 6^6^) node. The porous structure presents dimethylammonium cations in its cavities which are disordered over three different positions at room temperature since they sit on a twofold and a threefold axis. The phase transition at 155 K implies slight changes in the three-dimensional network and the loss of the two- and threefold axes of the dimethylammonium cations, giving rise to an enlarged unit cell with the *c* axis multiplied by three. The blocking of the counterions in the cavities seems to promote a change in the electric behaviour of compound (**1**), being paraelectric at room temperature and antiferroelectric at low temperature. Anomalies in the heat capacity and polarization measurements also suggest the onset of a ferroelectric phase below 3.5 K (Cañadillas-Delgado *et al.*, 2012 ▸; Guo *et al.*, 2017 ▸). The electric behaviour of this compound has been the subject of alternative explanations, such as the presence of spin-arrangement defects promoting a magnetoelectric coupling effect (Guo *et al.*, 2017 ▸) or a symmetry breaking compatible with a small polarization mainly from the off-centred Fe^III^—O octahedron (Tang *et al.*, 2019 ▸).

Regarding its magnetic behaviour, compound (**1**) exhibits negative magnetization and can be assigned as a Néel *N*-type ferrimagnet, with asymmetric magnetization reversal in the hysteresis loop. Long-range magnetic order appears below *ca* 37 K, and there is a compensation temperature (*T*_comp_) at 29 K. The magnetic structure has been determined through neutron diffraction experiments at 2, 17 and 33 K. Once magnetic order is reached, the Fe^II^ site (Fe1 in the 18*e* Wyckoff position) is coupled antiferromagnetically with Fe^III^ (Fe2 and Fe3 in the 6*b* and 12*c* Wyckoff positions, respectively) (see Fig. 1 ▸, bottom). Above *T*_comp,_ the magnetic moment on the Fe^II^ site is slightly higher than that of the Fe^III^ site, while the opposite occurs below *T*_comp_. The measurement of the magnetic moment on each site suggests that the Fe^II^ site orders first, with the magnetic moments oriented along the direction of the external magnetic field. This is consistent with a Néel *N*-type ferrimagnet with negative magnetization. The only model fitting the neutron data correctly is a ferrimagnet in which the magnetic moments are mainly oriented along the *c* axis, with a small component within the *ab* plane for the Fe^II^ site. At 2 K the magnetization value for the Fe^II^ site corresponds to 97.5% of the total saturation value, while for Fe^III^ it reaches only 82% of the total saturation value. The ferrimagnetic moment, arising because of the non-compensation of the Fe^II^ and Fe^III^ sublattices, calculated as the vector sum of the magnetic moments obtained from the neutron measurements at 2 K along the *c* axis, is 0.3 μ_B_ (Cañadillas-Delgado *et al.*, 2012 ▸). This value is slightly lower than that obtained from the magnetometry study by Zhao *et al.* (2010 ▸). It should be noted that these neutron diffraction experiments give information only about the magnetic moments resident on the iron sites and are not sensitive to the delocalized moments.

The combination of geometric distortion or strong covalence effects could be responsible for the difference between the values obtained from magnetometry measurements and neutron diffraction. Nevertheless, in order to analyse the magnitude of the magnetic moment over the magnetic and non-magnetic atoms, as well as the distribution of the spin density in the organic linkers, polarized neutron diffraction experiments are necessary.

Different questions also remain open about the electric behaviour of this compound and its analogues. One of them concerns the factors responsible for the structural phase transition at 155 K that gives rise to the antiferroelectric order. Different combinations of metals *M* ^II^/*M* ^III^ have been reported in the last few years and, seemingly, the disorder on the metal positions is a factor that contributes to suppressing the structural transition. Disorder of the metal sites is also proposed by Guo *et al.* (2017 ▸) as an explanation for the electric behaviour at low temperature: the increase in the dielectric constant below 50 K could originate from a ferroelectric order occurring due to spin-arrangement defects produced by Fe^II^/Fe^III^ disorder. Polarized neutron diffraction is well adapted to checking these hypotheses, since it offers a sensitivity superior to other techniques such as X-ray or unpolarized neutron diffraction for the study of this kind of disorder. Furthermore, a possible spin delocalization – that can only be detected by polarized neutron diffraction – could have implications for the magneto-electric behaviour. A possible delocalization towards the counterion may give an indication of the origin of the magneto-electric coupling, while a delocalization towards the oxygen atoms surrounding the magnetic ions could help to explain the observed behaviour at low temperature, in line with the mechanism proposed by Tang *et al.* (2019 ▸), since it may be related to a distortion of the coordination octahedron around the iron atoms that could give rise to electric polarization.

Therefore, with the aim of addressing the open questions mentioned above and contributing to the understanding of compound (**1**) and others related to it where similar mechanisms may be at play, the present work is dedicated to the analysis of the spin-density distribution in compound (**1**), by means of polarized neutron diffraction measurements using the flipping ratio technique (Bacon, 1975 ▸).

## 2. Materials and methods   

### 2.1. Neutron diffraction   

A suitable 1 × 1 × 3 mm single crystal was mounted on a vanadium pin on the four-circle D19 diffractometer at ILL, with a neutron wavelength of 1.16705 Å, equipped with a very large position-sensitive detector. Using a Displex cryo­refrigerator device we cooled the crystal down to 45 K at a cooling rate of 3 K min^−1^ to avoid any damage, and a full data set was collected.

### 2.2. Polarized neutron diffraction   

Flipping ratio measurements were performed with the same single crystal as used on D19, on the D3 polarized neutron diffractometer at ILL, with an incident wavelength of 0.84 Å. The orientation of the crystal was chosen with the *c* axis vertical, along the magnetic field. The flipping ratio measurements were done at 45 and 10 K, with the cooling rate of the sample being 3 K min^−1^ in a field of 9 T.

## 3. Results and discussion   

Polarized neutron diffraction measurements were performed on the D3 diffractometer at ILL, in normal beam geometry with a 9 T external magnetic field applied vertically along the *c* axis, to be sensitive to the magnetic moment contribution along this axis. The polarization on D3 is *p* = 0.94. The flipping ratios *R* of the most intense accessible reflections with magnetic contribution were collected at 45 and 10 K, above and below the temperature of the magnetic order transition (37 K).

**Figure 2:**
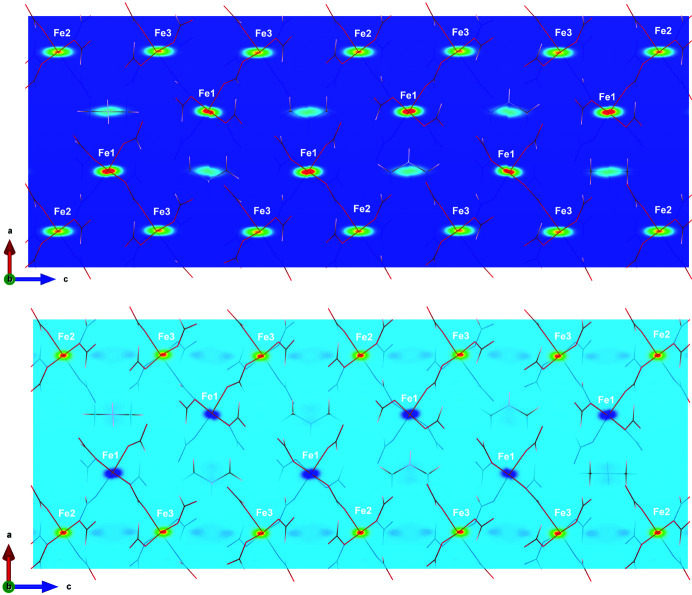
Views along the [010] direction of a section (*b* ≃ 0) of the spin-density maps obtained through the maximum entropy method at (top) 45 K and (bottom) 10 K. The spin-density maps were obtained with the crystal aligned with the *c* axis along the magnetic field. The colour scale denotes increasing spin density. At 45 K all iron sites show positive densities, while at 10 K positive densities correspond to the Fe2 and Fe3 sites (Fe^III^), and negative densities to Fe1 (Fe^II^).

Direct methods were used in order to obtain a first approach to the spin density. At 45 K, in the paramagnetic state, both the Fourier transform and the maximum entropy calculation show mainly not only dominant positive spin densities at the iron sites, but also a small positive contribution near the central nitro­gen atom of the counterion (N1), see Fig. 2 ▸. This contribution from the counterion is ten times smaller than the contribution from the iron sites for the maximum entropy map.

At 10 K, once magnetic order is achieved, the spin-density map from the maximum entropy method shows that the Fe^III^ (Fe2 and Fe3 atom sites) magnetic moment is oriented along the external magnetic field, with positive density, while the Fe^II^ (Fe1 atom site) magnetic moment is oriented antiparallel, with negative density. Nearly all the spin density is located on the iron sites, although along the *c* axis there is a weak negative density between the Fe^III^ sites, which is not located on any atom of the structural model. The Fourier transform shows not only the same disposition of the density at the iron sites, but also that there is a small positive delocalized density contribution near the oxygen atoms of the Fe^III^ environments. The maximum contribution on the oxygen atoms is about four times smaller than the positive density on the Fe^III^ sites. However, the density near the oxygen atoms decays much faster than the contribution on the iron sites, giving rise to a much smaller global spin-density contribution. At this temperature, no significant contribution is found near the counterions by any of the methods. Although both direct methods provide a reasonable starting point, the differences between them are also important. The Fourier transform is quite dependent on the *Q* range collected, and truncation effects due to the small number of flipping ratio reflections are common (this artificially affects a vanishing magnetic structure factor to all reflections in the considered *Q* range that were not actually measured). Maximum entropy analysis has been shown to give much more consistent results than conventional Fourier syntheses, by considerably reducing both noise and truncation effects (Papoular & Gillon, 1990*a* ▸,*b* ▸; Papoular *et al.*, 1995 ▸). For the sake of comparison both results are described in this article.

**Figure 3:**
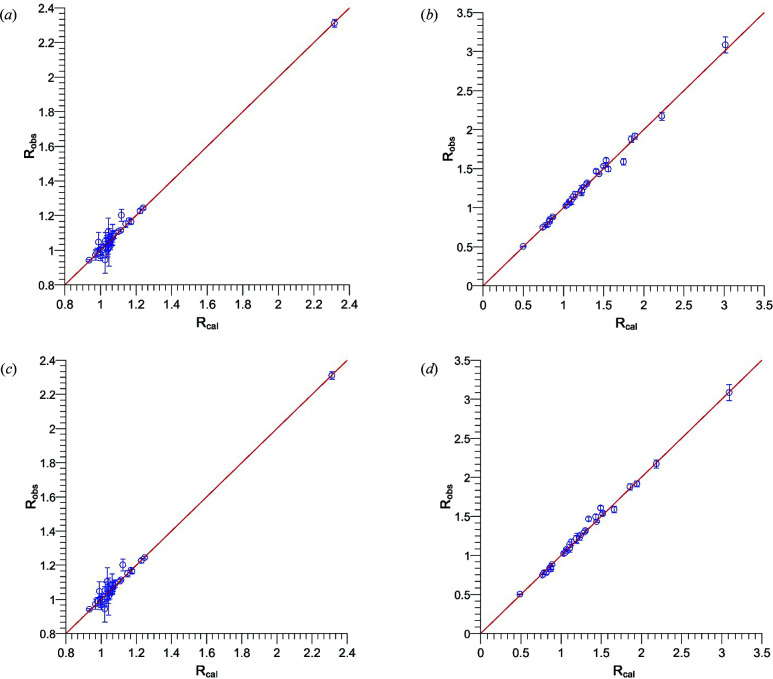
Observed *versus* calculated flipping ratios refined using the dipolar model (*a*) at 45 K (*R*_flip_ = 2.9%) and (*b*) at 10 K (*R*_flip_ = 1.9%), and using the multipolar approach (*c*) at 45 K (*R*_flip_ = 2.8%) and (*d*) 10 K (*R*_flip_ = 2.2%).

To describe the magnetic structure of compound (**1**) and distinguish between the spin μ_*S*_ and orbital μ_*L*_ components of the magnetic moments, we compared the measured flipping ratios with a model, using the dipolar approximation described in equation (15) in the mathematical background section (Appendix *A* [App appa]). A second approach was used to obtain a more sophisticated magnetic model, the multipolar approach. This method allows us to obtain a more precise spin-density model, which could be seen as a distorted model of the dipolar approach. The mathematical background of this approach can also be consulted in Appendix *A* [App appa]. The flipping ratios obtained on beamline D3 at two different temperatures were refined using *FullProf* (Rodríguez-Carvajal, 1993 ▸; Frontera & Rodríguez-Carvajal, 2004 ▸; the programs of the *FullProf* suite can be obtained at http://www.ill.eu/sites/fullprof). It is worth noting that the *FullProf* program calculates the magnetic structure factor in a simplified manner, since it supposes that only the component along the vertical axis contributes. The calculated *versus* observed flipping ratios obtained with each model above and below the order temperature are shown in Fig. 3 ▸.

In order to obtain the nuclear structure factors *F*_N_, an accurate structural model was determined at 45 K, in the paramagnetic phase, from single-crystal neutron diffraction on the D19 diffractometer (Cañadillas-Delgado *et al.*, 2012 ▸), using the same crystal as measured on D3. These results were fed into *FullProf* in order to obtain the nuclear structure factor *F*_N_ for *I*_+_ and *I*_−_, corrected by the sample extinction factors. During the refinements with dipolar and multipolar models, the iron(III) sites (Fe2 and Fe3) were constrained to have the same refined value, as shown in Tables 1 ▸ and 2 ▸.

In the measurement of the flipping ratio at 45 K, in the paramagnetic state, the observed signal is due to the field-induced magnetic moment and is in very good agreement with the value obtained from the magnetometry measurements at the same temperature [*ca* 1.3 (1) μ_B_ per formula unit]. It deserves to be noted that, in the paramagnetic phase, the observed magnetic signal on each atom is notably lower than in the ordered state. In this particular case, we can compare directly the results in the paramagnetic and in the ordered ground states. This is because the magnetic structure presents an antiferromagnetic coupling along the *c* axis and the applied magnetic field is low enough to prevent a spin-flop transition.

**Figure 4:**
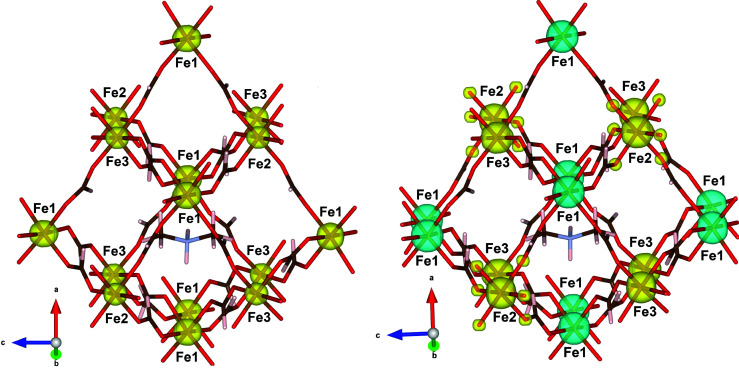
Spin-density maps obtained from the dipolar model (left) at 45 K and (right) at 10 K. Positive densities are shown in yellow and negative in blue.

The spin-density maps at 45 and 10 K were derived from the flipping ratios by refining the magnetic structure factors using a dipolar model and a multipole model; the latter model was limited to spherical terms due to the relatively low number of observations. The data refinement at 45 K gives a spin density located on the Fe^II^ and Fe^III^ positions. As expected in the paramagnetic state, the resulting moments on the iron sites Fe^II^ and Fe^III^ are parallel and positive (along the direction of the external magnetic field) (see Fig. 4 ▸, left). In contrast with the results of the direct methods, no significant spin density was found near the counterion or delocalized on the oxygen atoms of the iron environments, within experimental error. Moreover, the refinement using the dipolar approach shows that there is only a spin contribution (μ_*S*_), since the orbital contribution (μ_*L*_) is zero [see equation (15) in Appendix *A* [App appa]].

Below the magnetic order temperature, at 10 K the measurements indicate that almost all the spin density is located on the iron sites, with a small spin delocalization on atom O6 in the Fe2 environment (see Fig. 4 ▸, right). At this temperature, the Fe^II^ and Fe^III^ magnetic moments are antiparallel, and the small spin density located on the oxygen site around the Fe^III^ has the same sign, which is consistent with a small spin delocalization from Fe2 to O6. This density is transferred from Fe^III^ to the oxygen atom. Fe2 presents an octahedral coordination, with six oxygen atoms filling the octahedral positions occupied by O6 and the symmetry-related atoms (see Fig. 1 ▸, top). The delocalization of the spin density to the oxygen atoms seems favoured by the initially lower distortion of the Fe^III^ octahedron. At 45 K the geometric values ϕ and *s*/*h* are 59.5° and 1.23, respectively, for the Fe^II^ environment, while the Fe^III^ octahedron has the geometric values ϕ and *s*/*h* of 59.9° and 1.22, respectively, for both Fe2 and Fe3 ions, which are closer to the ideal values (ϕ = 60° and *s*/*h* = 1.22 for an ideal octahedron; Stiefel & Brown, 1972 ▸). Moreover, the environment of Fe2 is more regular than that of Fe3, keeping the same distance Fe—O in all the octahedra [Fe2—O6 is 2.0046 (10) Å, while Fe3—O2 and Fe3—O4 are 2.0200 (9) and 1.9913 (10) Å, respectively]. Yet the shift of spin density to the formate oxygens surrounding Fe2 observed at a lower temperature could imply a distortion of the octahedron, as discussed below.

The transfer of density is compatible with the moment reduction observed on Fe^III^ in comparison with Fe^II^. The refined magnetic moment on the Fe^II^ and Fe^III^ atoms corresponds to 90% and 79% of the ideal value considering *S* = 2 and 5/2, respectively. At 10 K, the experimental data allow us to refine the μ_*L*_ component in the dipolar refinement. The obtained results are larger than the experimental errors, and therefore we can conclude that there is a small orbital contribution to the magnetic moments of Fe^II^ and Fe^III^. The orbital contribution on the Fe^II^ atoms is two times larger than the contribution obtained on the Fe^III^ sites. It deserves to be noted that in the case of the free Fe^III^ ion system the orbital angular moment contribution is null, but in coordination polymers this value could be modified due to covalency. An alternative explanation for the orbital contribution observed on the Fe^III^ sites could be due to the mixture of Fe^III^ and Fe^II^ on the 6*b* Wyckoff position. In an isomorphic compound crystallized with different divalent metal atoms (*M* ^II^ = Zn, Ni and Mg), a significant mixture of Fe^III^/*M* ^II^ was reported on all metal sites by Ciupa *et al.* (2015*a* ▸) In their work, each metallic site was statistically distributed with equal probability between Fe^III^ and *M* ^II^ in the case of Ni and Zn, and *ca* 30:70 in the Mg case.

Disorder of metal sites was also proposed by Guo *et al.* (2017 ▸). In that study the authors suggest that the increase in the dielectric constant below 50 K, which is attributed to a ferroelectric order, occurs due to spin-arrangement defects promoting ferroelectricity. The influence of the counterion in the dielectric constant measurements may be negligible, as concluded from single-crystal measurements with the electric field parallel or perpendicular to the *c* axis. The values of the dielectric constant for the *E* || *c* configuration are significantly larger than for the perpendicular configurations, with no frequency dependence. The authors also point out that the polarization parallel to the *c* axis also changes depending on the crystal used in the measurements, which indicates the existence of defects. These defects could be produced by a random replacement of Fe^II^ ions on the Fe^III^ sites and *vice versa*, due to the requirements of charge balance.

Spin-arrangement defects among sites can be difficult to determine experimentally, in particular if the disorder among sites is less pronounced than that reported by Ciupa *et al.* (2015*a* ▸). Refinements using X-rays or unpolarized neutrons have less sensitivity, and neither the form factors of each site, on average, nor the metal–oxygen bond distances show a significant difference to obtain an accurate Fe^III^:*M* ^II^ ratio, either through least-squares refinement or through bond-valence calculation, respectively. Polarized neutrons can give us a much more accurate result, as shown by Plakhty *et al.* (1999 ▸), who presented the case of the inorganic compound Ca_3_Fe_2_Ge_3_O_12_, where the flipping ratio technique was able to detect a small contribution of 0.2% of Fe^II^ on the Fe^III^ position (Plakhty *et al.*, 1999 ▸). In our case, a refinement combining Fe^III^ and Fe^II^ on all sites was carried out. However, the values for divalent metal on the trivalent sites and *vice versa* were always below 0.05%, which is within the precision of our refinements, and they therefore have no physical meaning. Therefore, from the presented results, we consider that a well ordered compound is the correct assumption. The experimental values of the spin population after the last refinement are reported in Tables 1 ▸ (dipolar model) and 2 ▸ (multipolar model).

Although direct methods indicate that there could be a small spin contribution placed close to the dimethyl­ammonium counterion at 45 K, an improvement in the refinement is observed using indirect methods, via dipolar and multipolar approaches, with no significant magnetic contribution over the counterion molecule. Therefore, the weak contribution observed by direct methods near to the dimethyl­ammonium molecule should be attributed to truncation effects in the Fourier series.

At 10 K the appearance of a weak signal on the oxygen atoms of the Fe^III^ environments agrees with the map obtained from direct methods, where a weak positive signal is found on the oxygen atoms (O6) of the environment of one of the two crystallographically independent Fe^III^ ions in the structure. This density could be responsible for the slight difference between the sum of the magnetic moments in the magnetometry measurements and that from the non-polarized neutron diffraction data.

The ferroelectric signal observed at low temperature in this compound was explained by Tang *et al.* (2019 ▸), through density functional theory (DFT) calculations, by a symmetry breaking from 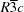 to *R*3*c* as a possible origin of the total polarization. Their study reveals that the small polarization originates mainly from the off-centred Fe^III^–O octahedron, and therefore has a structural origin. The obtained model provides polarization values larger than those observed experimentally. This discrepancy could come from the partial cancellation between the polarization arising from the iron octahedron and that from the counterion.

**Figure 5:**
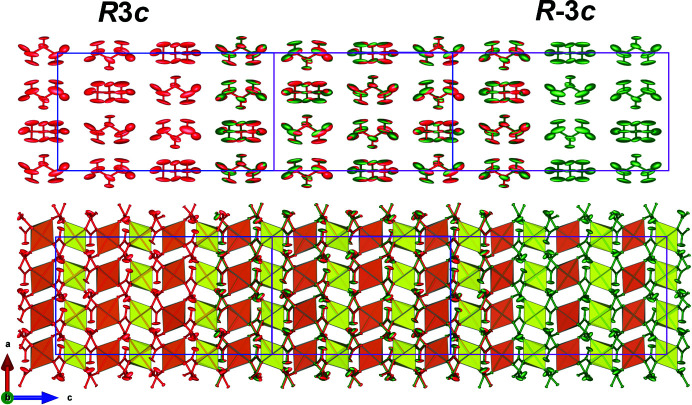
Views of a fragment of the crystal structure refined in space groups *R*3*c* and 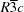 using the neutron diffraction data collected at 45 K. The *R*3*c* and 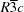 models are represented in red and green, respectively. The unit cells, represented in blue for *R*3*c* and pink for 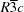, have been shifted to highlight the overlap between the two structures. For the sake of clarity, the upper figure only contains the counterions, while in the bottom figure only the 3D framework is shown.

Although these theoretical calculations suggest a structural model based on the *R*3*c* space group, our data refinement in the paramagnetic phase at 45 K does not provide any solid evidence of this breaking of symmetry. The refinement carried out in *R*3*c* using single-crystal neutron diffraction data provides statistics of *R*_1_ = 3.9%, *wR*_2_ = 6.7% and goodness of fit = 1.046, which are slightly better than the 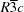 values (*R*_1_ = 4.5%, *wR*_2_ = 7.0% and goodness of fit = 1.088). The small improvement in the statistics of the *R*3*c* space group comes from the increase in the number of refinement parameters (170/337 for centro- and noncentrosymmetric space groups, respectively). However, the atomic positions in the *R*3*c* model present additional pseudo-symmetry in the structure and the anisotropic displacement parameters are close to having no physical meaning. The application of an inversion centre leaves the crystal structure invariant at 45 K (see Fig. 5 ▸). Therefore, our results suggest 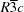 as the correct space group below the previously reported phase transition. However, with the current data we cannot discard the breaking of symmetry from 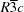 to *R*3*c* below 3.5 K, which is compatible with both specific-heat and polarization measurements (Cañadillas-Delgado *et al.*, 2012 ▸; Guo *et al.*, 2017 ▸). Moreover, the increase in spin delocalization at low temperature can produce slight structural changes, variations in the Fe—O bond distances or slight distortions of the metal environment. These subtle variations may be at the origin of the predicted symmetry breaking. To shed light on this point an accurate structural model below 3.5 K will be needed. The local magnetic moments, considering collinear spins coupled anti­ferro­magnetically, were also calculated by Tang *et al.* (2019 ▸). The calculated values of the spin moments of Fe^II^ and Fe^III^ ions were |3.7| μ_B_ and |4.3| μ_B_, respectively, which agree well with our results at 10 K [obtained considering the nuclear structure factor (*F_N_*) derived from the structural model obtained in the space group 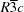].

## 4. Conclusions   

The magnetoelectric properties of the heterometallic compound [NH_2_(CH_3_)_2_][Fe^III^Fe^II^(HCOO)_6_] have been widely studied, not only with magnetic susceptibility and dielectric measurements but also with non-polarized neutron diffraction and DFT calculations. This compound is a nearly collinear *N*-type ferrimagnet where the magnetic moments of the Fe^II^ and Fe^III^ sites do not follow the same temperature/field dependence: the magnetization of the Fe^II^ site increases faster than that of the Fe^III^ sites. When a sufficiently large external field (5 T) is applied at 2 K, the magnetic moments of the Fe^III^ sites are oriented parallel and those of the Fe^II^ site antiparallel to the external field. The magnetic structure of this compound was determined by (non-polarized) neutron diffraction. The results revealed a low saturation value of the magnetic signal on the Fe^III^ sites and a ferromagnetic moment along the *c* axis lower than that obtained from the magnetometry study (see Table 3 ▸).

Measurements on the D3 instrument at ILL with polarized neutron diffraction permit us to analyse the magnetic moments not only at the iron sites but also at the non-metal sites, allowing an explanation of the low saturation on the Fe^III^ site. The measurements were done with an external field of 9 T at two different temperatures. At 45 K, in the paramagnetic state, the measurements reveal that the spins of both the Fe^II^ and Fe^III^ sites align along the external magnetic field, as expected, with a sum of about 1.32 (3) μ_B_ along the *c* axis, in good agreement with the magnetometry measurements. The spin-density maps do not reveal any significant magnetic contribution from the non-metallic atoms. At 10 K, within the magnetic ordered phase, the measurement shows the magnetic moments of the Fe^III^ sites oriented parallel to the external field, while the Fe^II^ site is oriented antiparallel, with values of about 3.96 (2) and −3.6 (3) μ_B_, respectively. At this temperature, there is also a small contribution of about 0.43 (7) μ_B_ from the O6 atom. This delocalization is only observed in one of the two environments of the Fe^III^ atoms, corresponding to the Fe2 site. All these contributions result in a sum of about 1.2 (3) μ_B_ along the *c* axis (see Table 3 ▸). It deserves to be noted that the ideal magnetic saturation, which corresponds to 5 and −4 μ_B_ for Fe^II^ and Fe^III^, respectively, would also give a sum of 1 μ_B_. From the data refinement, the models discard the occurrence of any spin density on the counterion sites. Accordingly, the sign of the spin moments and the sum of the vertical contributions correspond well with those expected from the magnetometry measurements and the theoretical calculations (Tang *et al.*, 2019 ▸).

The spin delocalization from Fe^III^ (Fe2) towards O6 that becomes noticeable at low temperature may be related to the reported magnetoelectric coupling of this compound (Guo *et al.*, 2017 ▸). The possible presence of spin-arrangement defects due to the mixture of Fe^III^/Fe^II^ that would be at the origin of the electric behaviour at low temperature was evaluated, but the data analysis discarded this scenario. Instead, the spin delocalization around Fe^III^ (Fe2) ions may imply a distortion of the Fe^III^—O bond distances that could be related to the ferroelectric signal, in a mechanism similar to the one proposed from DFT calculations (Tang *et al.*, 2019 ▸).

In conclusion, there is a non-negligible spin delocalization in this system below the Néel temperature. The contribution from the oxygen atoms that build the coordination environment of the Fe2 ions explains the origin of the low saturation found in the non-polarized neutron experiments. These results also explain why the magnetometry and unpolarized neutron diffraction measurements show a ferrimagnetic component with a non-compensated moment lower than the predicted value of 1.0 μ_B_ that should correspond to a Fe^II^–Fe^III^ antiferromagnetically coupled system. Further studies are needed to clarify the influence of spin delocalization on magnetoelectric coupling.

## Figures and Tables

**Figure 6:**
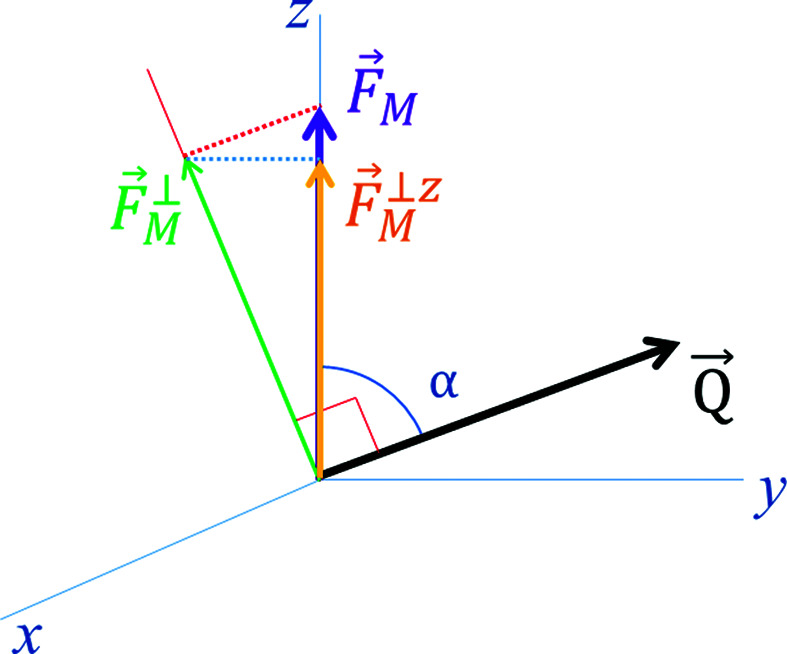
The disposition of the different components of the magnetic structure factor. The external magnetic field is applied along the *z* axis.

**Table:** μ_*T*_ = μ_*L*_ + μ_*S*_, where μ_*L*_ is the orbital contribution of the magnetic moment and μ_*S*_ is the spin contribution of the magnetic moment. For further details, see the mathematical background section in Appendix *A* [App appa].

Refinement details		
Magnetic field	9 T	9 T
Temperature	45 K	10 K
χ^2^	0.78	1.84
R_flip_ (%)	1.07	2.06

**Table:** 

	Local magnetic moments (μ_B_)					
	45 K			10 K		
	μ_*T*_	μ_*L*_	μ_*S*_	μ_*T*_	μ_*L*_	μ_*S*_
Fe^II^	0.92 (1)	∼0	0.92 (1)	−3.6 (3)	−0.8 (5)	−2.7 (5)
Fe^III†^	0.40 (1)	∼0	0.40 (1)	3.96 (2)	0.38 (12)	3.58 (12)
O1, O3, O5 (around Fe^II^)	∼0			∼0		
O6 (around Fe^III^)	∼0			0.43 (7)		0.43 (7)
O2, O4 (around Fe^III^)	∼0			∼0		
N1 (counterion)	∼0			∼0		
C1A (counterion)	∼0			∼0		
Sum	1.32 (3)			1.22 (32)		

† The magnetic moments of both Fe^III^ sites were constrained to be equal.

**Table:** 

Refinement details		
Magnetic field	9 T	9 T
Temperature	45 K	10 K
χ^2^	1.01	2.6
R_flip_ (%)	1.22	2.5

**Table:** 

Local magnetic moments (μ_B_)	μ_*T*_ at 45 K	μ_*T*_ at 10 K
Fe^II^	0.90 (10)	−3.59 (6)
Fe^III†^	0.39 (10)	4.00 (5)
O1, O3, O5 (around Fe^II^)	∼0	∼0
O6 (around Fe^III^)	∼0	0.30 (6)
O2, O4 (around Fe^III^)	∼0	∼0
N1 (counterion)	∼0	∼0
C1A (counterion)	∼0	∼0
Sum	1.29 (10)	1.01 (11)

† The magnetic moments of both Fe^III^ sites were constrained to be equal.

**Table:** The magnetization value at 2 K measured over a single crystal with H along the *c* axis was found to be 0.62 µ_B_ (Zhao *et al.*, 2010 ▸).

Magnetic moment components, in μ_B_, determined with non-polarized neutron diffraction at 2 K (Cañadillas-Delgado *et al.*, 2012 ▸)				
	*M_a_*	*M_b_*	*M_c_*	*M*_total_
Fe1 (Fe^II^)	0.6 (5)	−0.6 (5)	3.8 (3)	3.9 (4)
Fe2 (Fe^III^), Fe3 (Fe^III^)	0	0	−4.1 (2)	−4.1 (2)
Sum				−0.2 (4)

**Table:** 

Magnetic moments, in μ_B_, determined with polarized neutrons through the dipolar method at 10 K			
	μ_*T*_	μ_*L*_	μ_*S*_
Fe1 (Fe^II^)	−3.6 (3)	−0.8 (5)	−2.7 (5)
Fe2 (Fe^III^), Fe3 (Fe^III^)	3.96 (2)	0.38 (12)	3.58 (12)
O6 [around Fe2(Fe^III^)]	0.43 (7)		0.43 (7)
Sum	1.22 (32)		

**Table:** 

Magnetic moments, in μ_B_, determined with polarized neutrons through the multipolar method at 10 K	
	μ_*T*_
Fe1 (Fe^II^)	−3.59 (6)
Fe2 (Fe^III^), Fe3 (Fe^III^)	4.00 (5)
O6 [around Fe2(Fe^III^)]	0.30 (6)
Sum	1.01 (11)

## Appendix A Mathematical background

The flipping ratio method consists of measuring the ratio *R* between the Bragg intensities measured for incident neutron polarization parallel (‘spin up’, *I*_+_) or anti-parallel (‘spin down’, *I*_−_) to the applied magnetic field, to determine the magnetic structure factors experimentally,

The scattering cross section of the diffracted beam, , depends on the polarization of the incident beam, *p*, which is related to the efficacy of the instrument in polarizing the incident neutron beam and to the efficiency of the external magnetic field in maintaining the polarization over the whole trajectory,

where *n*_+_ represents the number of neutrons with spin up and *n*_−_ the number of neutrons with spin down, being in the ideal case |*p*| = 1. Equation (3) gives the incident polarization-dependent intensity:

In these equations *p* ^+^ and *p* ^−^ are the actual spin-up and spin-down polarizations of the incident beam. *F*_*N*_ is the nuclear structure factor, and can be obtained from a previous known nuclear model (atom positions and displacement parameters).  is the projection of the magnetic structure factor (**F**_*M*_) over the perpendicular to the scattering vector (**Q**), and  is the component of  which is parallel to the external applied beam (see Fig. 6 ▸). Since α is the angle between the scattering vector, **Q**, and the vertical axis, the relationship between these projections would be =  = .

In order to increase *F*_*M*_, large magnetic fields are needed if the system remains in a paramagnetic state. For ferromagnetic systems, the magnetic easy axis should be oriented with the external magnetic field. The application of an external magnetic field is necessary in this case to orient the different domains and to keep the neutron polarization during the experiment, so large magnetic fields are not needed.

Taking all of that into account, the flipping ratio in terms of the nuclear and magnetic structure factors is given by:

The magnetic structure factor can be determined solving the second-degree equation

with γ = *F_M_*/*F_N_*, *B* = −2[(*p* ^−^ *R* + *p* ^+^)/(*R* − 1)] and *C* = 1/sin^2^α. The resolution of this equation for non­centro­symmetric space groups becomes impossible, since the magnetic structure factor includes a non-real part, and thus two unknowns have to be obtained from the measurement of one flipping ratio. In that case a parametric model is required to obtain the spin-density map.

Based on equation (1), the intensities of the spin-up (*I*_+_) and spin-down (*I*_−_) neutrons are affected equally for sample absorption, therefore (to the first order) absorption corrections are not required in the flipping ratio analysis. However, other corrections are necessary during the data analysis. It is required to calculate an extinction factor for *I*_+_ and *I*_−_, and if the sample presents a high magnetic anisotropy, which can produce a depolarization of the beam, then the effective polarization of the incident beam should be corrected by a factor determined from the measurement of the polarization before and after the crystal. Moreover, at low temperatures, a high external magnetic field could polarize the nuclear spins, mainly in hydrogen atoms, and the atomic cross section would be different for spin-up and spin-down neutron polarization. Additionally, the Schwinger effect appears, due to the interaction between the neutron beam and the electric field produced by the nuclear and electric charges. This interaction gives rise to a contribution in the intensity for non-centrosymmetric structures. However, this effect is generally very weak and it is usually neglected.

The reconstruction of the spin-density map from the magnetic structure factor is performed through direct (Fourier transform and maximum entropy) or indirect methods (parametric models). The direct methods make no assumptions about the distribution of the spin density.

### A1. Direct methods: Fourier transform and maximum entropy methods

The Fourier transform consists of obtaining the spin-density map by means of the direct Fourier inversion of the scalar magnetic structure factors, *F*_*M*_,

where *M*(**r**) is the magnetization density. This method suffers from the limitations of the experimental measurement, since the obtained map is precise only if the sum is extended to all reciprocal-lattice vectors. If a large number of magnetic structure factors are not accessible, truncation effects are produced in the Fourier transform as spurious ripples. Experimentally, only those reflections with a large enough nuclear structure factor *F*_*N*_ contribution are accessible, whatever the magnetic structure factor magnitude. Thus the ripples affect the spin-density map. Moreover, in the Fourier transform all the magnetic structure factors have the same weight in the analysis, and the experimental uncertainties are not considered. The presence of incorrect magnetic structure factors due to spurious effects in the measurement of the flipping ratios will be directly transferred to the spin-density map. Finally, we can solve equation (6) only for centrosymmetric structures, since it is not possible to obtain the magnetic structure factors directly from the flipping ratios for a noncentrosymmetric structure.

An alternative method for obtaining the spin-density distribution is using the maximum entropy technique (Papoular & Gillon, 1990*a* ▸). This technique explores different spin-density maps, compatible with the experimental magnetic structure factors, and maximizes the entropy, trying to obtain χ^2^ = 1. The resulting spin-density map represents the most probable solution. The method is based on Bayes’ theorem, which defines the probability of a spin-density map being generated by the experimental data, *P*(map/data), as

In this equation *P*(data/map) is the probability of the data being those that generate a map which is supposed to be true. It is related to the agreement between the experimental and calculated magnetic structure factors that gives the spin-density map that is supposed to be true, which is χ^2^:

For *N* magnetic structure factors the expression of χ^2^ is

where  is the magnetic structure factor of the reflection **Q** calculated for the spin distribution ρ, and  is the magnetic structure factor obtained from the measured flipping ratio *R*.

*P*(map) is the likelihood of the spin-density map, whatever the magnetic structure factors are. The most probable map, with the highest value of *P*(map), is that which presents the highest entropy *S*:

where α is a constant related to the noise in the data.

Finally, *P*(data) is the probability of obtaining the data, and it is considered that *P*(data) = 1, since the data are known from the experiment.

### A2. Indirect methods: dipolar and multipolar models

The indirect methods (dipolar and multipolar models) consist of modelling the magnetic structure factors with an analytic expression that contains parameters that we obtain and refine from the experimental flipping ratios. This method attributes a magnetic moment to each atom site, and thus the structural symmetry is taken into account from the beginning. In order to obtain the spin-density maps we used the *FullProf* suite of programs to treat the magnetic structure factors with dipolar and multipolar models.

The dipolar model assumes that the spin-density distribution is described by the radial wavefunctions of the unpaired electrons of the atoms in the structure. The magnetic form factor, *f*(*s*), is approximated by a linear combination of the tabulated magnetic form factors of the free ions (*International Tables for Crystallography*, 2006 ▸). Using *s* = sinθ/λ the magnetic form factor is written as

The analytical approximation of the magnetic form factors 〈*j*_*l*_(*s*)〉 is a polynomial combination with three exponential terms as follows:

for *l* = 2, 4, 6. The coefficients *A*_*l*_, *a*_*l*_, *B*_*l*_, *b*_*l*_, *C*_*l*_, *c*_*l*_, *D*_*l*_, *A*_0_, *a*_0_, *B*_0_, *b*_0_, *C*_0_, *c*_0_ and *D*_0_ are tabulated for each magnetic atom (*International Tables for Crystallography*, 2006 ▸). The coefficients *W*_*l*_ are the variable parameters that *FullProf* refines and they can be related to the spin and orbital magnetic moments of the atom. Comparing equation (11) with the most common expression for the dipolar approximation [equation (14)], we can reformulate equation (11) considering only the lowest-order spherical harmonics (*l* = 0, 2),

As result, the expression of the magnetic form factor for the dipolar approximation can be written as

where the orbital contribution is μ_*L*_ = μ_*T*_ *C*_2_ = *W*_2_, and the spin contribution is μ_*S*_ = μ_*T*_ − μ_*L*_ = *W*_0_ − *W*_2_.

In the case of multipolar refinements, the used density approach considers the magnetic form factor as the Fourier transform of the magnetization density around an atom situated at the origin of the unit cell. This magnetization can be written as a sum of Slater-type functions,

In this expression, the unit vector **r**_0_ is defined along **r** and parametrized in terms of the spherical angles, *n*_*l*_ are the exponents of the Slater functions,  are real spherical harmonics defined in the literature (Kara & Kurki-Suonio, 1981 ▸),  are real coefficients and ζ_*l*_ are the volume factors. The spherical terms *s*_1_ and *s*_2_ account for a good *s–p* hybrid orbital simulation. With this model, *FullProf* refines the real coefficients and the volume factors can be adjusted. In our case, only the effective spherical coefficients () were considered and refined. The volume factors were set to the values tabulated by Clementi & Roetti (1974 ▸). The exponent of the Slater function was calculated from the orbital of the unpaired electrons for each atom. It was set to *n*_*s*1_ = 4 for the iron sites, and *n*_*s*1_ = 2 for oxygen and nitro­gen atoms.
